# Risk Factors of Vulvovaginal Candidiasis among Women of Reproductive Age in Xi'an: A Cross-Sectional Study

**DOI:** 10.1155/2018/9703754

**Published:** 2018-06-07

**Authors:** Xianling Zeng, Yafei Zhang, Taohong Zhang, Yan Xue, Huiqiu Xu, Ruifang An

**Affiliations:** ^1^Department of Obstetrics and Gynecology, The First Affiliated Hospital of Xi'an Jiaotong University, Xi'an, Shaanxi 710061, China; ^2^Department of General Surgery, The Second Affiliated Hospital of Xi'an Jiaotong University, Xi'an, Shaanxi 710004, China

## Abstract

*Purpose. *To explore risk factors of vulvovaginal candidiasis (VVC) among women of reproductive age in Xi'an district and then to offer reference for clinical prevention and treatment of VVC.* Methods. *Patients from the outpatient department of gynecology and obstetrics in the First Affiliated Hospital of Xi'an Jiaotong University from June 2016 to May 2017 were recruited strictly according to the inclusion and exclusion criteria. Participants diagnosed as simple VVC were assigned to the case group, while women who underwent routine gynecological examination and had normal vaginal microflora were assigned to the control group. Then we conducted a questionnaire survey of the two groups and used the logistic regression model to explore the related risk factors of VVC.* Results.* In the present study, ninety-seven cases were sample VVC patients and eighty-seven cases were healthy women. This cross-sectional study showed that occasionally or never drinking sweet drinks (odds ratio [OR] =0.161, 95% confidence interval [CI] =0.056-0.462, P=0.001), occasionally or never eating sweet foods (OR=0.158, 95%CI=0.054-0.460, P=0.001), and the use of condom (OR=0.265, 95%CI=0.243-0.526, P=0.001) were regarded as protective factors for VVC. In addition, sedentary life style (OR=7.876, 95%CI=1.818-34.109, P=0.006), frequently wearing tights (OR=6.613, 95%CI=1.369-27.751, P=0.018), frequent intravaginal douching (OR=3.493, 95%CI=1.379-8.847, P=0.008), having the first sexual encounter when under 20 years old (OR=2.364, 95%CI=1.181-7.758, P=0.006), the number of sexual partners being over two (OR=3.222, 95%CI=1.042-9.960, P=0.042), history of curettage (OR=3.471, 95%CI=1.317-9.148, P=0.012), history of vaginitis (OR=8.999, 95%CI=2.816-28.760, P<0.001), and not cleaning the vulva before or after sexual encounters (OR=13.684, 95%CI=2.843-65.874, P=0.001) were considered to be risk factors of VVC.* Conclusion. *In conclusion, risk factors of VVC are various, involving ages, hygienic habits, disease history, and other aspects.

## 1. Introduction

Vulvovaginal candidiasis (VVC) is the second most common vaginal infection affecting women of reproductive age, mainly damaging the vulva and vagina. It is estimated that approximately 70-75% of women of childbearing age will have at least one episode of VVC during their lifetime and 40-50% will suffer from a recurrence [1]. Almost 80-90% of VVC is caused by* Candida albicans* except that only a minority of cases (10-20%) is caused by* non-C. albicans* species, usually* Candida glabrata* [2].* Candida albicans* is part of normal vaginal microflora. It will become a robust opportunistic fungal pathogen and is the leading causative agent of VVC when the body lacks protective immunity and has recalcitrance to clearance [3]. Moreover, the anus is close to the vagina anatomically, providing much convenience for the migration of gut organisms, including Candida, to the vagina. VVC gives rise to much untold discomfort in many patients, poses a threatening problem to clinicians, and generates considerable direct and indirect economic costs associated with medication and health-care visits. Apart from the above negative impacts, some emerging data also have suggested that VVC during pregnancy might be associated with increased risk of pregnancy complications, such as premature rupture of membranes, preterm labor, chorioamnionitis, and congenital cutaneous candidiasis [4, 5]. At the same time, there was a potential relationship between VVC and preterm birth [6]. In addition, several studies have suggested that VVC could contribute substantially to enhanced susceptibility to HIV infection [7, 8]. However, the etiology and pathogenesis of VVC are not clear. According to present epidemiological studies, the prevalence and incidence of reproductive tract infections vary between countries and ethnicities [9, 10]. Even in similar population groups, the epidemiological features of the low reproductive tract infections are different [11]. The reasons for the difference in the incidence of VVC among women of childbearing age from various races and regions are still unknown and the risk factors for VVC remain unclear. Therefore, it is necessary to analyze the risk factors of VVC among women of reproductive age in Xi'an district and then to offer reference for clinical prevention and treatment of VVC.

## 2. Methods

### 2.1. Subjects

This cross-sectional study was carried out in Gynecology Outpatient Clinic of the First Affiliated Hospital of Xi'an Jiaotong University from June 2016 to May 2017. Inclusion criteria are the following: (1) being 18-50 years old with a history of sexual life; (2) being diagnosed with simple VVC or normal vaginal microflora; (3) menstruation ending since at least 3 days, no vaginal irrigation or drug delivery in the vagina within 7 days, and no sexual intercourse within 3 days before examination. Exclusion criteria are as follows: women who had mixed vaginitis; women who were in the period of pregnancy, menstruation, or lactation; women who had hysterectomy or bilateral excision of the appendix. Besides, patients with incomplete medical record were also excluded. Simultaneously, we selected those women who underwent routine gynecological examination and had normal vaginal microflora as the control group. After obtaining each interviewee's written informed consent, we conducted a face to face questionnaire survey of the two groups and analyzed the potential risk factors of VVC. The study protocol was performed with approval of the ethics committee of the First Affiliated Hospital of Xi'an Jiaotong University.

### 2.2. Diagnostic Criteria

The normal vaginal microflora was defined as follows: the vaginal flora's density was grade II-III, the vaginal flora's diversity was grade II-III, the dominant flora was Lactobacillus, the vaginal pH was 3.8-4.5, the function of Lactobacillus was normal, and the leukocyte esterase was negative. The diagnosis of VVC requires pelvic examination and laboratory tests. The combination of erythema, edema of vulvar and vaginal tissues, and thick white vaginal discharge suggests the diagnosis of VVC. Furthermore, if the pH of vaginal secretion is less than 4.5, sprout spores and pseudohyphae are found under the oil microscope, and the Gram staining is positive, then it should be reported as VVC.

### 2.3. Sampling and Tests

The patient was in the bladder lithotomy position. One researcher put a speculum lubricated by saline into the vagina, swabbed a bit of vaginal secretion by a dry cotton, coated it on the slide from left to right evenly, and put the cotton swab into a clean tube with little saline. Then, the researcher scraped vaginal secretion by another dry cotton and put the cotton swab into a clean dry tube. The sample was tested for morphological examination and functional detection by professional laboratory technicians within 30 minutes. The former included density of vaginal flora, diversity of vaginal flora, the dominant flora, and the detection of pathogenic microorganism, while the latter contained the value of pH and the activity of various metabolites and enzymes, as well as markers of inflammatory reaction. All the samples were collected by the same researcher and tested by two experienced inspectors at the same time. If the results of the two inspectors were inconsistent, we resorted to a third veteran for the final diagnosis.

### 2.4. Investigation of Risk Factors

The questionnaire was designed according to epidemiological methods. The contents included general information such as age, residence, occupation, and educational level; daily living habits such as the frequency of drinking sweet drinks, the frequency of eating sweet foods, the frequency of physical training, sedentary life style, and daily emotional state; hygienic habits such as menstrual care, underwear material and the frequency of alternation, and the forward direction of wiping (forward wiping); previous history and reproductive history such as marriage state, history of vaginitis, and history of curettage; sexual behaviors such as the age at first sexual intercourse, number of sexual partners, frequency of sexual life, and so on.

### 2.5. Statistical Analysis

Data was analyzed though SPSS 20.0, and P<0.05 was considered to be of great statistical difference. Univariate analysis was used to calculate crude odds ratios (OR) and 95% confidence intervals (CI). A multiple logistic regression model was used to control confounding factors and to determine which factor still remained statistically significant. The factor with great statistical difference after univariate analysis was entered into multivariate logistic regression analysis.

## 3. Results

### 3.1. Univariate Analysis of General Information and Daily Living Habits

Eventually, ninety-seven patients with VVC and eighty-seven women who underwent routine gynecological examination and had normal vaginal microflora were recruited strictly according to the inclusion and exclusion criteria. They all were at the age of 18-50 and had a regular menstruation. Univariate analysis showed that age under 40 years old was a risk factor for VVC (OR=2.431, 95%CI=1.061-5.568, P=0.032). While the residence, occupation, and educational level posed almost no effect on the occurrence of VVC (P>0.05) (see Table 1). By the way, frequently drinking sweet drinks (OR=2.960, 95%CI=1.483-5.907, P=0.002) and eating sweet foods (OR=0. 2.685, 95%CI=2.442-9.839, P=0.039), occasionally or never performing physical training (OR=3.418, 95%CI=1.272-9.182, P=0.011), sedentary life style (OR=2.954, 95%CI=1.515-5.759, P=0.001), and a distressed emotional state (OR=3.191, 95%CI=1.729-5.890, P<0.001) were regarded as risk factors for VVC with great statistical differences (see Table 1).

### 3.2. Univariate Analysis of Hygienic Habits

The present study revealed that nonuse of pads during the nonmenstrual period (OR=0.387, 95%CI=0.093-0.501, P<0.001) and occasional or never wearing tights (OR=0.351, 95%CI=0.153-0.805, P=0.011) could help to lessen the risk of VVC. Yet wiping direction after the toilet, frequency of underwear replacement, and underwear material exerted no effect on the susceptibility of VVC (P>0.05) (see in Table 1).

### 3.3. Univariate Analysis of Previous History, Reproductive History, and Sexual Behavior

This cross-sectional study indicated that no history of vaginitis (OR=0.273, 95%CI=0.143-0.566, P=0.001), occasional or never intravaginal douching (OR=0.455, 95%CI=0.218-0.953, P=0.030), no history of curettage (OR=0.306, 95%CI=0.167-0.561, P<0.001), the use of condom (OR=0.382, 95%CI=0.199-0.730, P= 0.003), single sexual partner (OR=0.461, 95%CI=0.215-0.986, P=0.037), and cleaning the vulva before or after sexual life (OR=0.473, 95%CI=0.230-0.972, P=0.039) were favorable factors for avoiding suffering VVC. However, having the first sexual encounter when being under 20 years old (OR=4.007, 95%CI=1.714-9.371, P<0.001) would increase the risk of VVC. At the same time, the analysis of marriage state, the frequency of cleaning the vulva, having sexual life during menstruation, and the frequency of sexual encounters showed no relation with VVC's risk (see in Table 2).

### 3.4. Multivariate Logistic Regression Analysis

Multivariate logistic regression analysis revealed that occasionally or never drinking sweet drinks (OR=0.161, 95%CI=0.056-0.462, P=0.001), occasionally or never eating sweet foods (OR=0.158, 95%CI=0.054-0.460, P=0.001), and the use of condom (OR=0.265, 95%CI=0.243-0.526, P=0.001) were regarded as protective factors for VVC. In addition, sedentary life style (OR=7.876, 95%CI=1.818-34.109, P=0.006), frequently wearing tights (OR=6.613, 95%CI=1.369-27.751, P=0.018), frequent intravaginal douching (OR=3.493, 95%CI=1.379-8.847, P=0.008), having the first sexual encounter when under 20 years old (OR=2.364, 95%CI=1.181-7.758, P=0.006), the number of sexual partners being over two (OR=3.222, 95%CI=1.042-9.960, P=0.042), history of curettage (OR=3.471, 95%CI=1.317-9.148, P=0.012), history of vaginitis (OR=8.999, 95%CI=2.816-28.760, P<0.001), and not cleaning the vulva before or after sexual life (OR=13.684, 95%CI=2.843-65.874, P=0.001) were considered to be risk factors of VVC (see in Table 3).

## 4. Discussion

VVC is a universal health problem affecting millions of women and is caused by excessive growth of yeasts in the vaginal mucosa [12]. It could cause various vaginal signs and symptoms, including a thick cottage-cheese-like discharge associated with vaginal and vulvar pruritus, pain, burning, erythema, and edema. External dysuria and dyspareunia may also occur.

This study showed that women younger than 40 years old had a twofold higher risk of getting VVC than the elder. Moreover, age at first sexual intercourse less than 20 increased the risk of suffering VVC by four times. Even in multivariate logistic regression analysis model, this trend still exited. Coincidently, an epidemiological study described an increased Candida vaginitis in women at reproductive age rather than those at menopause [13]. The reason for VVC being more common in young women may lie in the fact that they are easy to suffer from adverse factors such as risky sexual behaviors [14]. Besides, some physiological and tissue changes, caused by reproductive hormones, which happen in women during this stage of life, increase susceptibility to Candida infection. In present study, residence, occupation, educational background, and marriage status showed no impact on the occurrence of VVC. However, a previous study indicated that symptomatic episodes of VVC were significantly correlated with employed women [15]. Another study discovered that married women had a higher risk of suffering VVC than the single, while high education could protect against the infection [16]. The divergence may be explained by the fact that there was almost no difference between rural areas and urban districts with increasing awareness of self-care and improvement of medical conditions in current times. At the same time, people of different educational backgrounds could have the same avenue to obtain the massive information related to health-care through the Internet. Studies [17–19] have showed that diabetes mellitus, especially uncontrolled diabetes mellitus, was conducive to the occurrence of VVC. The current study could not elute the relation between diabetes mellitus and VVC because of lack of data on diabetes mellitus. But this study explored that frequently drinking sweet drinks and frequently eating sweet foods could augment the susceptibility to VVC. This was the first time to take dietary habits into consideration. Even in multivariate logistic regression analysis, occasionally or never drinking sweet drinks and occasionally or never eating sweet foods showed a protective effect. This may contribute to the fact that increased glucose concentrations in vaginal secretions could promote the adherence of Candida to epithelial cells and further stimulate its development.

This study identified frequently wearing tights as a risk factor for VVC, while there was no association between frequency of underwear replacement and underwear material and VVC. A recent study found that risk factors for VVC involved synthetic underclothing, frequently wearing tight pants, and so on [20]. Another study showed that type of underwear (cotton/synthetic) was statistically associated with the presence of recurrent VVC [21]. This probably is attributed to the fact that wearing tight clothes seems to foster friction and maceration, thereby increasing the local acidity and therefore the fungal infection. This research revealed that history of curettage increased possibility of Candida infection, which was consistent with a previous study which found that abortive women had a high proportion of infection than women without abortion [16]. The study detected that condom was a protective factor helping to prevent against VVC. It was a pity that this study classified ligation, intrauterine device (IUD), oral contraceptives (OCP), and so on as one category for which only three women took OCP regularly. So it was difficult to identify the effect of certain type on the occurrence of VVC. Similarly, Dou et al. [16] also found no relation between OCP and VVC. However, a study reported that there was a link between the IUD use and VVC [22]. Therefore, the role of IUD in the development of VCC is still unclear, needing further investigation. This study showed that frequent intravaginal douching was an adverse factor of women, which was in line with the result of previous studies [15, 16]. The reason may lie in that intravaginal practices could cause damage to vaginal and rectal tissues and disrupt the vaginal flora as well. Moreover, intravaginal douching may disturb the balance of vaginal microecosystem, lead to the decline of vaginal homeostasis, encourage the growth of yeasts, and further cause VVC. In contrast to this, one study hold that the association between intravaginal practices and VVC was not definite [23]. At the same time, this study showed that frequency of cleaning the vulva exerted no effect on the occurrence of VVC, while cleaning the vulva before or after sexual life was an advantageous factor for preventing against VVC. There was no study exploring the relation between the two before. Usage of pad during nonmenstruation was a risk factor for VVC, but the adverse effect disappeared in multivariate logistic regression analysis.

Fixed sexual partner could decrease the risk of suffering VVC. An observational study reported that a sexual partner was only associated with asymptomatic colonization [24]. At the same time, this study probed whether sexual life during menstruation or frequency of sexual life would have an effect on the occurrence of VVC. The result did not found definite association. Women who had a previous history of vaginitis were more likely to suffer VVC. This finding was concordant with some previous epidemiological studies which believed that there was an association between symptomatic episodes of VVC and a history of lower genital tract infection [15, 25, 26]. Besides, the study paid attention to the effect of doing regular exercise, sedentary life, and daily emotional state. Eventually, doing regular exercise and positive emotion were protective factors in univariate analysis, yet the beneficial effect disappeared when entering multivariate logistic regression analysis, While sedentary life was still a risk factor, increasing the susceptibility to VVC almost 8-fold. To date, little is currently known about the correlation between physical exercises and emotional factors and the prevalence of VVC.

The diagnostic criteria used in this study were microscopic examination in conjunction with clinical manifestations. First of all, compared with the microscopy methodology, fungal culture has a low specificity although it has a much better sensitivity, because one or a variety of* non-albicans Candida* species may reside in the vagina [27]. Next, the cure of VVC is marked by the control of symptoms, rather than the eradication of all Candida organisms from the genital system, which is also virtually impossible, at least in the long term. Hence, culture-independent tests cause much difficulty in treatment [28]. Importantly, according to* Expert consensus on the clinical application of vaginal microecological evaluation*, fungal culture is eventually used only when recurrent VVC occurs or multiple microscopic examinations are negative. In line with this, Dovnik et al. also suggested that the diagnosis of VVC is most frequently made clinically, in combination with microscopic examination of the discharge. When no fungal elements are identified under microscopy and no typical clinical signs are present, a woman is not likely to have VVC [29]. In this study, the objectives were patients with simple VVC for the first time, so fungal culture was not needed.

A shortage of the study was the relatively small sample size with limited small number of women diagnosed with VVC. Additionally, the study was an epidemiological survey which focused only on some outstanding challenges faced by clinicians. So it did not incorporate all potential risk factors like hypoimmunity, long-term use of antibiotic, pregnancy, hyperestrogenemia, hormonal imbalance, hyperglycemia, OCP, IUD, and so on [1]. What is more, the reports of dietary and other factors could not be supported by diaries or health records; hence recollection bias may have affected results. Certainly, this study was an in-depth cross-sectional study focusing on the potential risk factors of VVC. It included various aspects ranging from general information and daily living habits to hygienic habits and sexual behaviors. What is important is that the first-visit gynecological physician and the pathologists were the same persons throughout the survey, thereby warranting the consistency of the research.

In conclusion, occasionally or never drinking sweet drinks, occasionally or never eating sweet foods, and the use of condom were regarded as protective factors for VVC. In addition, sedentary life style, frequent wearing tights, frequent intravaginal douching, having the first sexual encounter when being under 20 years old, the number of sexual partners being over two, history of curettage, history of vaginitis, and not cleaning the vulva before or after sexual life were considered to be risk factors of VVC. Obviously, VVC poses a great threat to women's reproductive health. Risk factors of VVC are various, involving ages, hygienic habits, disease history, and other aspects. It is necessary to take corresponding measures to avoid risk factors and to help lessen the prevalence of VVC among women of reproductive age.

## Figures and Tables

**Table 1:** Univariate analysis of socioeconomic factors and daily living habits among women of reproductive age.

Clinical parameters	Total (n=184)	Case (n=97)	Control (n=87)	OR (95% CI)	P
Age					
<40 y	155	87	68	2.431(1.061-5.568)	**0.032 **
≥40 y	29	10	19	1.000	
Residence					
Rural area	53	32	21	0.646(0.338-1.236)	0.187
Town and city	131	65	66	1.000	
Occupation					
Farmer	34	21	13	1.592(0.737-3.436)	0.237
Employment	135	68	67	1.126(0.387-3.280)	0.828
Unemployment	15	8	7	1.000	
Educational background					
Secondary or below	75	42	33	1.250(0.692-2.256)	0.460
College or above	109	55	54	1.000	
Drinking sweet drinks					
Frequently	52	37	15	2.960(1.483-5.907)	**0.002 **
Occasionally or never	132	60	72	1.000	
Eating sweet foods					
Frequently	61	47	14	2.685(2.442-9.839)	**0.039 **
Occasionally or never	123	50	73	1.000	
Doing regular exercise					
Occasionally or never	162	91	71	3.418(1.272-9.182)	**0.011 **
Frequently	22	6	16	1.000	
Sedentary life					
Yes	131	79	52	2.954(1.515-5.759)	**0.001 **
No	53	18	35	1.000	
Daily emotional state					
Negative emotions (nervousness, anxiety, etc.)	109	70	39	3.191(1.729-5.890)	**<0.001**
Comfort	75	27	48	1.000	
Usage of pad during nonmenstruation					
No	145	66	79	0.387(0.093-0.501)	**<0.001**
Yes	39	31	8	1.000	
Wiping direction after the toilet					
Forward wiping	62	35	27	1.296(0.431-1.474)	0.311
Backward wiping	122	62	80	1.000	
Frequency of underwear replacement					
Over one day	10	6	4	1.500(0.199-2.681)	0.530
Less than one day	174	91	83	1.000	
Frequency of wearing tights					
Occasionally or never	151	73	78	0.351(0.153-0.805)	**0.011**
Frequently	33	24	9	1.000	
Underwear material					
Others	43	25	19	1.316(0.407-1.592)	0.367
Pure cotton	140	72	68	1.000	

OR: odds ratios; CI: confidence interval; y: year; Case, patients with simple VVC; control, women with normal vaginal microflora.

**Table 2:** Univariate analysis of previous history, reproductive history, and sexual behaviors among women of reproductive age.

Clinical parameters	Total (n=184)	Case (n=97)	Control (n=87)	OR (95% CI)	P
History of vaginitis					
No	128	56	72	0.273(0.143-0.566)	**0.001**
Yes	56	41	15	1.000	
Marriage status					
Single	43	25	18	1.043(0.668-2.654)	0.801
Married	141	72	69	1.000	
Frequency of intravaginal douching					
Occasionally or never	144	70	74	0.455 (0.218-0.953)	**0.030**
Frequently	40	27	13	1.000	
Frequency of cleaning the vulva					
More than three days	42	21	21	1.000(0.578-2.293)	0.999
Less than two days	142	76	66	1.000	
History of curettage					
No	97	38	59	0.306(0.167-0.561)	**<0.001**
Yes	87	59	28	1.000	
Contraceptive methods					
Condom	124	56	68	0.382 (0.199-0.730)	**0.003**
Others (ligation, IUD, and so on)	60	41	19	1.000	
Age at first sexual intercourse					
≤20 y	36	28	8	4.007(1.714-9.371)	**<0.001**
>20 y	148	69	79	1.000	
Sexual life during menstruation					
Yes	22	14	8	1.623 (0.645-4.084)	0.300
No	160	83	77	1.000	
Number of sexual partners					
1	147	72	75	0.461(0.215-0.986)	**0.037**
≥2	37	25	12	1.000	
Frequency of sexual life					
More than twice a week	127	65	62	1.280(0.437-1.535)	0.355
Less than once a week	57	32	25	1.000	
Cleaning the vulva before or after sexual life					
Yes	142	69	73	0.473(0.230-0.972)	**0.039**
No	42	28	14	1.000	

IUD: intrauterine device; y: year; OR: odds ratios; CI: confidence interval; Case, patients with simple VVC; control, women with normal vaginal microflora.

**Table 3:** Multivariate logistic regression analysis of risk factors for VVC among women of reproductive age.

Clinical parameters	*β*	SE	Wald	P	OR	95% CI
Age						
<40 y						
≥40 y	-1.263	0.691	3.347	0.067	0.283	0.073-1.094
Doing regular exercise						
Occasionally or never						
Frequently	-1.179	0.782	2.274	0.132	0.308	0.066-1.124
Sedentary life						
No						
Yes	2.064	0.748	7.615	**0.006 **	7.876	1.818-34.109
Daily emotional state						
Negative emotions (nervousness, anxiety, etc.)						
Comfort	0.633	0.5	1.601	0.206	1.884	0.706-5.023
Frequency of drinking sweet drinks						
Frequently						
Occasionally or never	-1.827	0.538	11.528	**0.001 **	0.161	0.056-0.462
Frequency of eating sweet foods						
Frequently						
Occasionally or never	-1.848	0.546	11.442	**0.001 **	0.158	0.054-0.460
Usage of pad during non- menstruation						
No						
Yes	1.18	0.617	3.653	0.056	3.255	0.970-10.918
Frequency of wearing tights						
Occasionally or never						
Frequently	1.819	0.768	5.612	**0.018 **	6.613	1.369-27.751
Frequency of intravaginal douching						
Occasionally or never						
Frequently	1.251	0.474	6.957	**0.008 **	3.493	1.379-8.847
Age at first sexual intercourse						
<20 y						
≤20 y	2.515	0.908	7.671	**0.006 **	2.364	1.181-7.758
Contraceptive methods						
Others (ligation, IUD, and so on)						
Condom	2.676	0.818	10.703	**0.001 **	0.265	0.243-0.526
Number of sexual partners						
1						
≥2	1.827	0.576	4.128	**0.042 **	3.222	1.042-9.960
History of curettage						
No						
Yes	1.245	0.494	6.336	**0.012 **	3.471	1.317-9.148
History of vaginitis						
No						
Yes	2.197	0.593	13.735	**<0.001**	8.999	2.816-28.760
Cleaning the vulva before or after sexual life						
Yes						
No	2.616	0.802	10.647	**0.001 **	13.684	2.843-65.874

IUD: intrauterine device; y: year; OR: odds ratios; CI: confidence interval.

## Data Availability

The data used to support the findings of this study are available from the corresponding author upon request.

## References

[1] Gonçalves B., Ferreira C., Alves C. T., Henriques M., Azeredo J., Silva S. (2016). Vulvovaginal candidiasis: epidemiology, microbiology and risk factors. *Critical Reviews in Microbiology*.

[2] Sobel J. D. (2007). Vulvovaginal candidosis. *The Lancet*.

[3] Peters B. M., Yano J., Noverr M. C., Fidel P. L. (2014). Candida vaginitis: when opportunism knocks, the host responds. *PLoS Pathogens*.

[4] Mølgaard-Nielsen D., Svanström H., Melbye M., Hviid A., Pasternak B. (2016). Association between use of oral fluconazole during pregnancy and risk of spontaneous abortion and stillbirth. *Journal of the American Medical Association*.

[5] Meizoso T., Rivera T., Fernández-Aceñero M. J., Mestre M. J., Garrido M., Garaulet C. (2008). Intrauterine candidiasis: report of four cases. *Archives of Gynecology and Obstetrics*.

[6] Roberts C. L., Morris J. M., Rickard K. R. (2011). Protocol for a randomised controlled trial of treatment of asymptomatic candidiasis for the prevention of preterm birth [ACTRN12610000607077]. *BMC Pregnancy and Childbirth*.

[7] Ray A., Ray S., George A. T., Swaminathan N. (2011). Interventions for prevention and treatment of vulvovaginal candidiasis in women with HIV infection. *Cochrane Database of Systematic Reviews*.

[8] Simon V., Ho D. D., Abdool Karim Q. (2006). HIV/AIDS epidemiology, pathogenesis, prevention, and treatment. *The Lancet*.

[9] Hamad M., Kazandji N., Awadallah S., Allam H. (2014). Prevalence and epidemiological characteristics of vaginal candidiasis in the UAE. *Mycoses*.

[10] Allsworth J. E., Peipert J. F. (2007). Prevalence of bacterial vaginosis: 2001–2004 national health and nutrition examination survey data. *Obstetrics & Gynecology*.

[11] Borman A. M., Szekely A., Linton C. J., Palmer M. D., Brown P., Johnson E. M. (2013). Epidemiology, antifungal susceptibility, and pathogenicity of *Candida africana* isolates from the United Kingdom. *Journal of Clinical Microbiology*.

[12] Ilkit M., Guzel A. B. (2011). The epidemiology, pathogenesis, and diagnosis of vulvovaginal candidosis: A mycological perspective. *Critical Reviews in Microbiology*.

[13] Holland J., Young M. L., Lee O., Chen S. C. A. (2003). Vulvovaginal carriage of yeasts other than Candida albicans. *Sexually Transmitted Infections*.

[14] Vaca M., Guadalupe I., Erazo S. (2010). High prevalence of bacterial vaginosis in adolescent girls in a tropical area of Ecuador. *BJOG: An International Journal of Obstetrics & Gynaecology*.

[15] Amouri I., Sellami H., Borji N. (2011). Epidemiological survey of vulvovaginal candidosis in Sfax, Tunisia. *Mycoses*.

[16] Na D., Weiping L., Enfeng Z., Chan W., Zhaozhao X., Honghui Z. (2014). Risk factors for Candida infection of the genital tract in the tropics. *African Health Sciences*.

[17] Curran J., Hayward J., Sellers E., Dean H. (2011). Severe vulvovaginitis as a presenting problem of type 2 diabetes in adolescent girls: A case series. *Pediatrics*.

[18] Nyirjesy P., Zhao Y., Ways K., Usiskin K. (2012). Evaluation of vulvovaginal symptoms and Candida colonization in women with type 2 diabetes mellitus treated with canagliflozin, a sodium glucose co-transporter 2 inhibitor. *Current Medical Research and Opinion*.

[19] Lattif A. A. (2011). Molecular typing and in vitro fluconazole susceptibility of *Candida* species isolated from diabetic and nondiabetic women with vulvovaginal candidiasis in India. *Journal of Microbiology, Immunology and Infection*.

[20] Ogouyèmi-Hounto A., Adisso S., Djamal J. (2014). Place of vulvovaginal candidiasis in the lower genital tract infections and associated risk factors among women in Benin. *Journal de Mycologie Médicale*.

[21] D'Antuono A., Baldi E., Bellavista S., Banzola N., Zauli S., Patrizi A. (2012). Use of Dermasilk briefs in recurrent vulvovaginal candidosis: Safety and effectiveness. *Mycoses*.

[22] Chassot F., Negri M. F. N., Svidzinski A. E. (2008). Can intrauterine contraceptive devices be a Candida albicans reservoir?. *Contraception*.

[23] Brown J. M., Hess K. L., Brown S., Murphy C., Waldman A. L., Hezareh M. (2013). Intravaginal practices and risk of bacterial vaginosis and candidiasis infection among a cohort of women in the United States. *Obstetrics & Gynecology*.

[24] Watson C. J., Fairley C. K., Grando D., Garland S. M., Myers S. P., Pirotta M. (2013). Associations with asymptomatic colonization with candida in women reporting past vaginal candidiasis: an observational study. *European Journal of Obstetrics & Gynecology and Reproductive Biology*.

[25] Giraldo P., Von Nowaskonski A., Gomes F. A. M., Linhares I., Neves N. A., Witkin S. S. (2000). Vaginal colonization by Candida in asymptomatic women with and without a history of recurrent vulvovaginal candidiasis. *Obstetrics & Gynecology*.

[26] Patel D. A., Gillespie B., Sobel J. D. (2004). Risk factors for recurrent vulvovaginal candidiasis in women receiving maintenance antifungal therapy: Results of a prospective cohort study. *American Journal of Obstetrics & Gynecology*.

[27] Blostein F., Levin-Sparenberg E., Wagner J., Foxman B. (2017). Recurrent vulvovaginal candidiasis. *Annals of Epidemiology*.

[28] Donders G. G. G., Sobel J. D. (2017). Candida vulvovaginitis: a store with a buttery and a show window. *Mycoses*.

[29] Dovnik A., Golle A., Novak D., Arko D., Takač I. (2015). Treatment of vulvovaginal candidiasis: a review of the literature. *Acta Dermatovenerologica Alpina, Pannonica et Adriatica*.

